# Aerobic Interval Training Regulated SIRT3 Attenuates High-Fat-Diet-Associated Cognitive Dysfunction

**DOI:** 10.1155/2018/2708491

**Published:** 2018-03-22

**Authors:** Zhaoling Shi, Chen Li, Yue Yin, Zheng Yang, Han Xue, Nan Mu, Yishi Wang, Manling Liu, Heng Ma

**Affiliations:** ^1^Department of Paediatrics, Xijing Hospital, Fourth Military Medical University, Xi'an, China; ^2^Department of Physiology and Pathophysiology, School of Basic Medicine, Fourth Military Medical University, Xi'an, China

## Abstract

Cognitive dysfunction is an important complicated disease in obesity. Exercise ameliorates obesity and the related cognitive dysfunction. However, the underlying mechanism is still unclear. In this study, we investigated whether aerobic interval training (AIT) could attenuate high-fat-diet- (HFD-) associated cognitive dysfunction and the possible mechanism of SIRT3-MnSOD pathway. C57BL/6 wild-type (WT) mice and SIRT3 knockout (KO) mice were randomized into control (Con) or HFD group with or without AIT training for 6 weeks. The spatial learning and memory ability were impaired in HFD group compared to the control group. The levels of mitochondrial protein acetylation were increased in the hippocampus of HFD group. The acetylation level of antioxidative MnSOD was increased as well. As a result, the ROS and MDA levels were significantly increased, which leads to the neuron apoptosis in the hippocampus. SIRT3 deficiency further aggravated HFD-induced cognitive dysfunction and susceptibility to oxidative stress injury. However, AIT upregulated neuron SIRT3 expression and decreased the acetylation of MnSOD. The hippocampus neuron oxidative stress and apoptosis were both decreased compared to untrained HFD group, which finally improved cognitive function of HFD mice. Collectively, AIT attenuates HFD-associated cognitive dysfunction through SIRT3 upregulation and improvement of antioxidative MnSOD activity.

## 1. Introduction

High-fat diet (HFD) is one of the main factors of obesity in modern society [1]. Obesity, as a metabolic disease, manifests the excessive fat accumulation and increased body weight. Obesity is strongly associated with various diseases, such as diabetes, hypertension, cardiovascular and cerebrovascular diseases, and cancer [2]. In rodents, HFD-induced obesity reduces hippocampus plasticity [3] and compromises spatial learning skill [4]. Recent studies indicated that obesity is associated with cognitive decline and neurodegenerative diseases [5].The rising epidemical evidence also shows that obesity is closely related to brain dysfunction and early onset of Alzheimer's disease [6]. It is important to understand the molecular mechanisms of HFD-associated cognitive dysfunction which may help to find therapeutic and prevention strategies to mitigate brain dysfunction in obesity.

Exercise training has been recognized to exert beneficial effects in obese individuals. Interestingly, clinical studies and animal experiments have shown that exercise training not only reduces body weight but also ameliorates brain dysfunction [7]. However, the majority of population failed to reach the recommended ≥150 minutes per week of moderate-intensity exercise. Aerobic interval training (AIT) is an emerging exercise approach, which typically involves repeated bouts of high-intensity exercise and interspersed with periods of low intensity recovery or rest. AIT exhibits similar cardiovascular and metabolic benefits as traditional moderate-intensity exercise [8]. Therefore, AIT may provide an effective and time-efficient alternative to improve physical condition. However, it remains unclear whether AIT would protect against cognitive dysfunction in HFD individuals.

The mitochondrial sirtuin SIRT3 is the major deacetylase that controls mitochondrial biological functions, including ATP generation, reactive oxygen species (ROS) detoxification, and mitochondrial dynamics. Therefore, SIRT3 has been demonstrated to play important roles in many neuronal physiological and pathological conditions [9]. In the nervous system, mitochondria SIRT3 deficiency would affect the normal function of the neuron [10]. Interestingly, research also indicated that aerobic training can upregulate SIRT3 expression [11]. We speculate that SIRT3 might have potential link in AIT and cognitive function.

In the present study, we newly found that AIT can effectively improve HFD-related cognitive dysfunction. SIRT3 is an important regulator that mediates neuroprotective effect of AIT in HFD individual via the antioxidative MnSOD in hippocampus neurons. There results may provide novel insight into exercise therapy in HFD-induced cognitive dysfunction.

## 2. Material and Methods

### 2.1. Animals and Reagents

All animal experiments were approved by Animal Ethical Experimentation Committee of the Fourth Military Medical University. Male C57BL/6 WT mice were obtained from the Animal Center of Fourth Military Medical University. SIRT3 KO (Sirt3^−^/^−^) mice in C57BL/6 background were established by K&D gene technology (Wuhan, China). Two-month-old adult male C57 WT mice and SIRT3 KO mice were used. Mice were randomly assigned to groups and fed with control diet group (Con: 6% kcal fat, 27% kcal protein, and 57% from carbohydrate) or high-fat diet group (HFD: 45% kcal fat, 20% kcal protein, and 35% kcal carbohydrate) for 12 weeks [12]. Mice were kept in a temperature-controlled house with 12-hour light-dark cycles so that they would perform the training protocol in darkness (active period). Throughout the trial, mice had free access to water and food. The body weight and the random-fed blood glucose were measured weekly at the same time point. A 100 *μ*l blood sample was collected from the tail vein to test the random-fed blood glucose of mice.

### 2.2. Aerobic Intermittent Training (AIT)

AIT was performed at 6 weeks and continued another 6 weeks as previously described [13, 14]. Aerobic capacity was assessed as VO_2max_ with a metabolic chamber equipped with a treadmill. All sessions were performed during the dark cycle of the animals (active period). After 1 week of adaptive training (10 m/min, 30 min/day, 5 days/week), mice were treated with AIT, which consisted of 10 min warm-up with 40%–50% of VO_2max_ and 4 min high-intensity running with 80–85% of VO_2max_ and interspersed by 2 min active rest (at speed of 12 m/min and corresponding to 65–70% of VO_2max_). After the last exercise session, the Morris water maze test was carried out to evaluate mice spatial learning and memory.

### 2.3. Morris Water Maze Test

The Morris water maze (MWM) test was used to evaluate spatial learning and memory of mice [15]. The latency time to escape and locate the platform in water maze was recorded as an index of acquisition or learning using a computer tracking system with EthoVision software (Noldus Information Technology, Wageningen, Netherlands). At the end of MWM test, mice would be decapitated and brain tissue was obtained immediately after the integral experiment. The right hippocampus of every mouse was separated, flash-frozen, and then stored at −80°C for subsequent experiments. And the left hippocampus was frozen in liquid nitrogen-cooled isopentane and sliced into 16-*μ*m-thick sections for subsequent TUNEL staining to detect apoptosis.

### 2.4. Measurement of ROS Generation and MDA Levels

The production of ROS in hippocampus samples was measured by General Oxidative Stress Indicator (#C6827, Thermo Fisher Scientific). Briefly, the hippocampus samples were labeled with the ROS sensitive fluorescent probes (CM-H_2_DCFDA), and the fluorescence intensity was observed under fluorescence microscope to reflect the changes of intracellular ROS content. The MDA concentration was measured by Lipid Peroxidation MDA Assay Kit (#S0131, Beyotime, China) according to the manufacturer's instructions. The results were expressed in nmol MDA/g protein.

### 2.5. Measurement of Mitochondrial MnSOD Activity

The mitochondrial MnSOD activity of hippocampus samples was measured using the Superoxide Dismutase Assay Kit (#706002-480, Cayman Chemical) according to the manufacturer's instructions

### 2.6. Western Blotting and Coimmunoprecipitation

Western blotting and coimmunoprecipitation (Co-IP) analysis were performed as previously described [16]. The mitochondria extraction was conducted under the protocol of the tissue mitochondria isolation kit (#C3606, Beyotime, China). Rabbit anti-SIRT3 (#2627), rabbit anti-COX IV (#4850), rabbit anti-GAPDH (#5174), rabbit anti-acetylated-lysine (#9441) antibody, mouse anti-*β*-Actin (#3700), and rabbit anti-GST (#2625) were purchased from Cell Signaling Technology (CST, USA). GST-tagged recombinant human SIRT3 protein (ab54333) and rabbit anti-MnSOD (ab13533) were purchased from Abcam. The data were analyzed using Quantity One software (Bio-Rad Laboratories, Inc. USA).

### 2.7. TUNEL Detection

In situ apoptosis was analyzed using a TUNEL Apoptosis Assay Kit following the manufacturer's instructions (#C1086, Beyotime, China). The hippocampal tissues were frozen in liquid nitrogen-cooled isopentane and sliced into 16-*μ*m-thick sections. The sections were fixed with 4% paraformaldehyde (PFA), washed with PBS, and incubated with 0.5% Triton X-100 (in PBS). The sections were then washed, incubated with the TUNEL solution, and observed with the Leica fluorescent microscope. A quantitative analysis of the percentages of viable neurons was performed using Image-Pro Plus (Leica DMLB) software at 400x magnification.

### 2.8. Cell Culture

Hippocampal neurons were obtained from the fetal mice (E16-E18). After trypsin digestion for 15 min in Hank's solution, the hippocampal neurons were washed and resuspended with DMEM (10% FBS). The hippocampal neurons were then plated and incubated in poly-L-lysine coated culture wells for 4 h. After the attachment, medium was replaced with neuronal culture medium (neurobasal medium supplemented with 2% B27, 0.5 mM glutamine, 25 mM glutamic acid). The hippocampal neurons were freshly prepared and incubated with different concentration of H_2_O_2_ with special neurobasal medium for 30 min. The cell culture reagents were purchased from Invitrogen.

### 2.9. Statistical Analysis

Quantitative data are were presented as mean ± SEM. Differences were compared by Student's *t*-test or 2-way ANOVA test with Turkey's multiple comparisons test correction. A 0.05 level of confidence was considered as statistically significant. The analysis was performed with the GraphPad Prism software version 5.0 (GraphPad Software, San Diego, CA).

## 3. Results

### 3.1. SIRT3 Deficiency Aggravates HFD-Induced Cognitive Dysfunction

**Figure 1:**
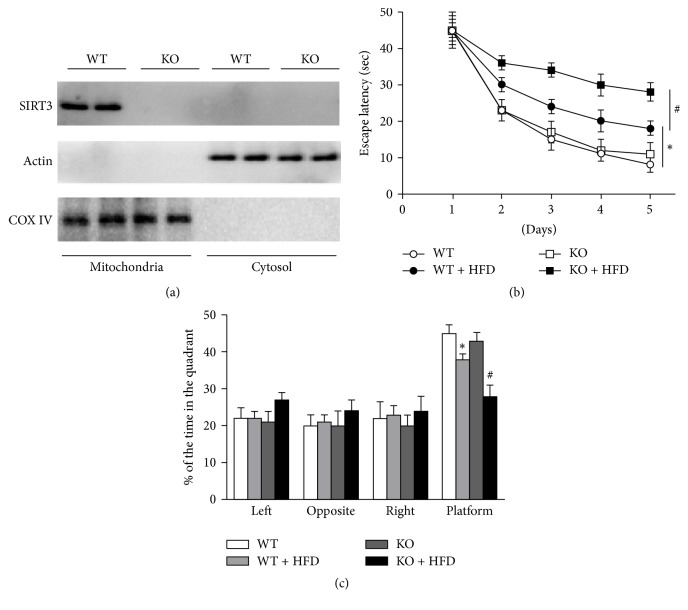
*SIRT3 deficiency aggravates HFD-induced cognitive dysfunction*. (a) Representative immunoblots of mitochondria and cytosol SIRT3 were detected by Western blots. WT and SIRT3 KO mice with or without HFD were subjected to 6-week AIT. (b) Escape latency of place navigation test. (c) Staying time in the quadrant in space probe test. ^*∗*^*P* < 0.05 versus WT; ^#^*P* < 0.05 versus HFD. Values are means ± SEM, *n* = 6 independent experiments.

The SIRT3 was located in the mitochondria and is highly expressed in the nervous system. We first demonstrated that the hippocampal SIRT3 of SIRT3 KO mice was completely knocked out (Figure 1(a)). After the HFD model was successfully established, we observed the spatial memory and learning ability of mice in each group. There was no difference in the escape latency between WT and SIRT3 KO mice with normal diets. Compared with the normal diet group, the escape latency of HFD mice was significantly prolonged in the place navigation test (Figure 1(b)). In the space probe test, the target quadrant retention time of HFD mice was significantly reduced (Figure 1(c)). Meanwhile, the spatial learning and memory functions in HFD-SIRT3 KO mice were seriously declined (Figures 1(b) and 1(c)). These data indicated that high-fat diet leads to impaired cognitive function, which is more evident in the SIRT3 deficiency mice.

### 3.2. HFD-Induced MnSOD Acetylated Inactivation and Elevated Oxidative Stress Levels in Hippocampus

**Figure 2:**
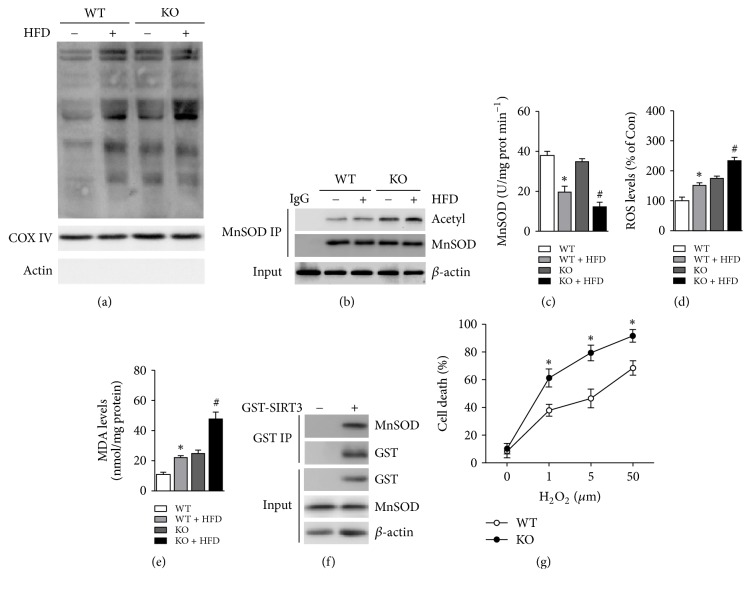
*HFD impairs SIRT3-MnSOD and increases hippocampal oxidative stress*. (a) The acetylation level of mitochondria protein was examined by immunoblots. (b) The acetylation of MnSOD was examined by coimmunoprecipitation. (c) MnSOD activity. (d) ROS levels in hippocampus. (e) mda levels in hippocampus. (f) The combination of SIRT3 and MnSOD was performed in vitro. (g) Cell death of hippocampal neurons with H_2_O_2_ treatment in vitro. ^*∗*^*P* < 0.05 versus WT; ^#^*P* < 0.05 versus HFD. Values are means ± SEM, *n* = 6 independent experiments.

SIRT3 regulates the cellular energy metabolism and oxidative stress through control of the deacetylation level in mitochondrial proteins. We detected the acetylation level of total mitochondrial proteins in the hippocampus of each group and found that HFD treatment significantly increased the acetylation level of mitochondrial proteins (Figure 2(a)). Given MnSOD is an important antioxidant enzyme in mitochondria. The binding relationship between SIRT3 and MnSOD was demonstrated by in vitro experiments (Figure 2(f)). We proposed that HFD may restrain the activity of MnSOD through acetylation modification and increase oxidative stress levels. The acetylation level of MnSOD was then examined by CO-IP experiments. The results showed that the acetylation of MnSOD was increased in HFD group and elevated in HFD-SIRT3 KO group (Figure 2(b)). Meanwhile, we detect the MnSOD activity and observed that the activity of MnSOD in HFD group was decreased (Figure 2(c)), which suggested that hippocampal MnSOD was acetylated inactivation. We next detected the levels of ROS and MDA in the hippocampus. The levels of ROS and MDA in the hippocampal neurons of HFD were significantly increased (Figures 2(d) and 2(e)). Notably, SIRT3 deficiency markedly exacerbated the oxidative stress after HFD treatment (Figures 2(d) and 2(e)). To further investigate the role of oxidative stress in hippocampal neurons injury, the hippocampal neurons were subjected to different concentrations of H_2_O_2_ in vitro. The cell death was significantly increased with elevated concentration of H_2_O_2_. Moreover, the cell viability in SIRT3 KO group was much lower compared to WT neurons, suggesting a key role of SIRT3 against oxidative stress in hippocampal neurons.

### 3.3. AIT Upregulates Hippocampal SIRT3-MnSOD Pathway

**Figure 3:**
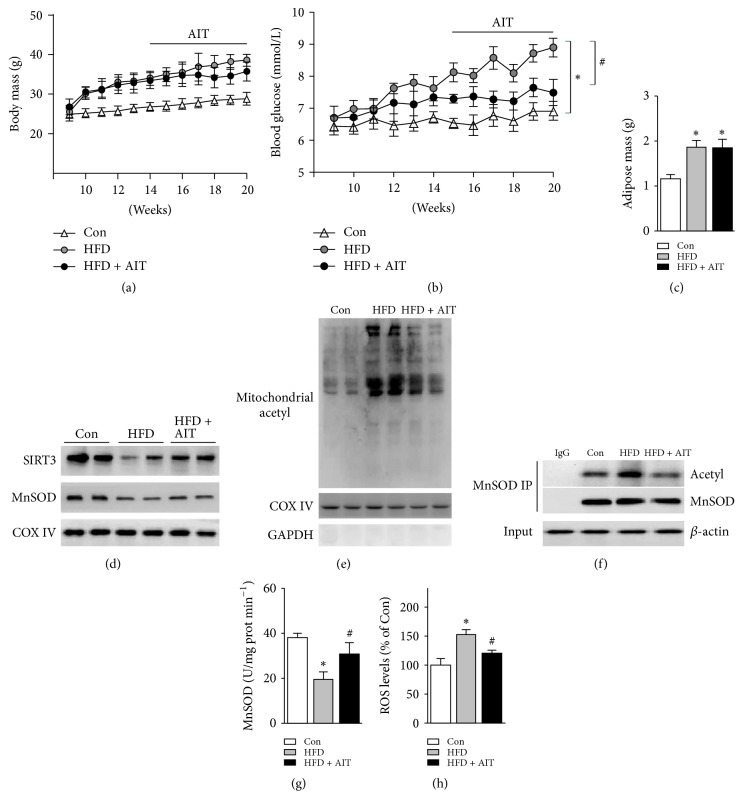
*AIT improves hippocampal SIRT3-MnSOD in HFD mice*. (a) Body mass of each group's mice. (b) Random-fed blood glucose of each group' mice. (c) Adipose mass of each group' mice. (d) Representative immunoblots of mitochondria SIRT3 and MnSOD were detected by Western blots. (e) The acetylation level of mitochondria protein was examined by immunoblots. (f) The acetylation of MnSOD was examined by coimmunoprecipitation. (g) MnSOD activity. (h) ROS levels in hippocampus. ^*∗*^*P* < 0.05 versus Con; ^#^*P* < 0.05 versus HFD. Values are means ± SEM, *n* = 6 independent experiments.

In order to clarify the effect of AIT in the HFD-induced cognitive impairment, mice were treated with AIT for another 6 weeks. We observed the body weight and the random-fed blood glucose of mice in each group weekly and found that AIT training failed to reduce body weight (Figure 3(a)), but the random-fed blood glucose had been significantly decreased (Figure 3(b)). At the same time, adipose tissue was measured after AIT. We found that adipose tissue mass was increased significantly in HFD group, but there was no significant difference between HFD-AIT and HFD group (Figure 3(c)). We further examined the expression of SIRT3 and MnSOD. The expression of SIRT3 and MnSOD was significantly reduced in HFD, while AIT significantly increased the expression of SIRT3 and MnSOD (Figure 3(d)). Similarly, we observed that the acetylation level of hippocampal mitochondrial proteins was decreased significantly after AIT treatment (Figure 3(e)). The acetylated modification of MnSOD was decreased (Figure 3(f)) while the MnSOD activity was significantly improved (Figure 3(g)). The level of ROS was decreased in HFD-AIT group compared to HFD group (Figure 3(h)). Collectively, these data suggested that AIT may decrease hippocampal oxidative stress through improving hippocampal SIRT3-MnSOD signaling. What is more, this effect is independent of weight loss.

### 3.4. AIT Inhibits Hippocampal Neuron Apoptosis and Improves the Cognitive Function of HFD Mice

**Figure 4:**
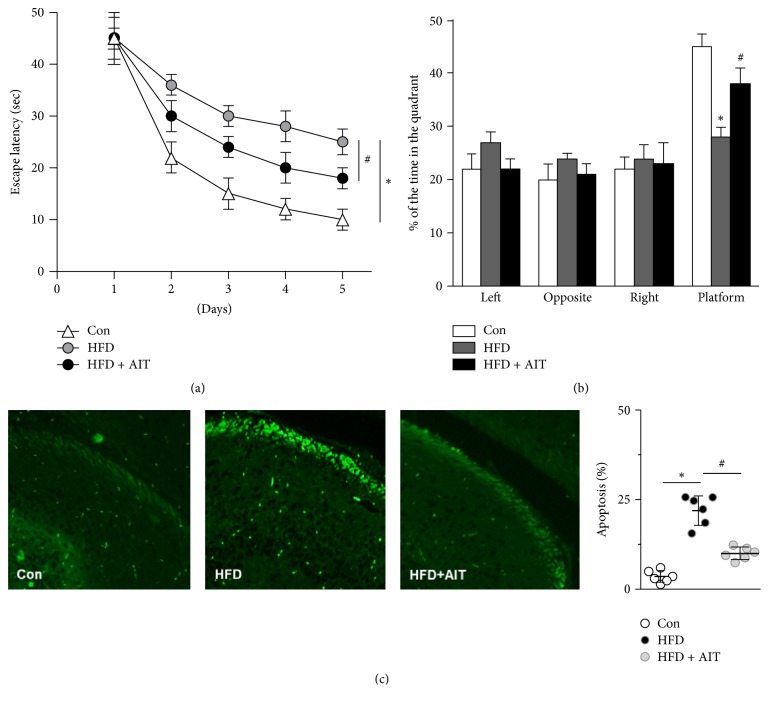
*AIT inhibits hippocampal neuron apoptosis and alleviates the cognitive dysfunction in HFD mice.* (a) Escape latency of place navigation test. (b) Staying time in the quadrant in space probe test. (c) Hippocampus slices of each group were subjected to TUNEL staining to detect apoptosis. ^*∗*^*P* < 0.05 versus Con; ^#^*P* < 0.05 versus HFD. Values are means ± SEM, *n* = 6 independent experiments.

The Morris water maze test showed that HFD seriously impaired the spatial cognition and memory ability of mice, as proved above. We subsequently analyzed if AIT could ameliorate HFD cognitive dysfunction. HFD mice were subjected to 6-week AIT. The escape latency was decreased and the target quadrant retention time was significantly increased in the HFD-AIT group (Figures 4(a) and 4(b)). These results indicated that high-fat diet decreases the spatial learning and memory ability of obese mice, which could be significantly reversed by AIT. As terminally differentiated cells, hippocampal neurons are very sensitive to oxidative stress. We next observed the apoptosis in hippocampal neurons with TUNEL staining. The data implied that the neuronal apoptosis was markedly increased in HFD group. Finally, AIT training reduced neuronal apoptosis compared to HFD group (Figure 4(c)). Taken together, these experiments indicated that AIT improves the spatial cognition and memory ability of HFD mice through reducing oxidative stress and neural apoptosis.

## 4. Discussions

In the present study, our findings suggest that HFD impairs hippocampal SIRT3-MnSOD pathway and cognitive function in mice. We newly demonstrate that the antioxidant effect of SIRT3-MnSOD is critical to neuron survival. AIT could attenuate neuronal apoptosis and improve cognitive function in HFD mice through upregulating SIRT3-MnSOD pathway and decreasing oxidative stress levels. Our study implied that SIRT3 may be a new factor in neuroprotective field.

Obesity, as a chronic metabolic disease has increasingly endangered people's health. High-fat diet is one of the main risk factors of obesity. Numerous studies show that obesity is closely related to cognitive dysfunction and memory loss [5]. Animal experiments also showed that high-fat diet for 8 weeks can increase hypothalamic neuronal apoptosis [17], increase the MDA content in serum and hippocampus tissue, and aggravate brain lipid peroxidation [18]. Clinical studies also show that obesity is negatively related to cognitive ability and brain function in individual [19]. Thus, it is very important to elucidate the mechanism of HFD-induced cognitive impairment.

Hippocampus is the representative area of learning and memory, which is closely related to cognitive function. Obesity would damage hippocampal neuron and further impair individual's cognitive function [20]. Neurons are particularly sensitive to oxidative injury. Obesity enhanced the neuronal fatty acid metabolism and ROS production, which leads to the neuronal membrane peroxidation and neuron apoptosis. Our research again confirms that HFD-induced obesity can increase oxidative stress and apoptosis in hippocampal neurons, which impairs the cognitive function in mice. SIRT3 is an important mitochondria deacetylase which conducts the antioxidant function by deacetylation of MnSOD. The level of SIRT3 activity and the expression of MnSOD were decreased in both diabetic and obese people [21]. However, the role of SIRT3 in HFD-induced cognitive dysfunction remains unclear. Our study further confirms that HFD-induced obesity and HFD-induced hippocampal neuronal damage are mainly due to decreased SIRT3-MnSOD antioxidative system.

Aerobic interval training (AIT), a new mode of sports training, is suitable for various people. Recent studies showed that AIT not only enhanced the heart and lung functions, but also had therapeutic effects on Alzheimer's disease and cognitive impairment [22]. Exercise training can improve the plasticity of hippocampus and reduced neuronal apoptosis [23]. More importantly, exercise training enhances the long term potentiation effect (LTP), which therefore improved the ability of learning and memory [15]. It was also proved that AIT increases the MnSOD activity and reduces the MDA level [24]. Clinical studies also proved that 45 minutes of daily physical training can increase cognitive ability in obese youth [25]. In this study, we found that AIT may decrease blood glucose, improve the spatial memory dysfunction, and reduce the ROS levels.

As the major deacetylase in mitochondria, SIRT3 controls the energy metabolism in neurons. SIRT3 enhances the antioxidant function of MnSOD by deacetylation [26]. However, high-fat diet can inhibit the expression of SIRT3 [27]. To further explore the role of SIRT3 in HFD-induced cognitive dysfunction, we further used SIRT3 KO mice to test whether SIRT3 is essential for the neuroprotective effects in HFD-induced hippocampal injury. Our results indicated that SIRT3 KO seriously aggravates the cognitive function and ROS levels in HFD mice. However, AIT effectively upregulates SIRT3 and MnSOD expression in the hippocampus neurons.

Collectively, our study demonstrated that AIT protects the hippocampus neurons from apoptosis and attenuates cognitive dysfunction. SIRT3 and the downstream MnSOD may be the key mechanism in the neuroprotective effects of AIT on HFD individuals. Our research provides new evidence for the mechanism of HFD-induced cognitive dysfunction and suggests that AIT may be a new therapeutic intervention.
